# Treatment of recalcitrant disseminated ulcerative necrobiosis lipoidica with ustekinumab

**DOI:** 10.1016/j.jdcr.2023.06.040

**Published:** 2023-07-07

**Authors:** Patrick McMullan, Aziz Khan, Jonas A. Adalsteinsson, Reid Waldman, Campbell Stewart, Brett Sloan, Marti Rothe

**Affiliations:** a Department of Dermatology, University of Connecticut School of Medicine, Farmington, Connecticut; b Department of Dermatology, University of Utah School of Medicine, Salt Lake City, Utah; c ICAHN School of Medicine at Mount Sinai, New York City, New York; d Dermatology Associates of Glastonbury, Glastonbury, Connecticut

**Keywords:** azathioprine, granulomatous dermatitis, necrobiosis lipoidica, ustekinumab

## Introduction

Necrobiosis lipoidica (NL) is an inflammatory granulomatous dermatitis (GD) that typically presents as a collection of chronic indurated and atrophic skin plaques most frequently on the lower extremities.^1^ Histologically, NL is characterized by the presence of collagen degeneration, sclerosis, and palisading granulomas within the dermis and subcutaneous tissue that occur as a result of immune complex deposition within the dermal vasculature.^1^ Clinical management of NL is a significant challenge because of its chronic and nonhealing nature, its propensity to ulcerate, and koebernize.^1^ Here, we report a case of severely treatment-resistant disseminated ulcerative NL on the trunk and extremities that was successfully managed using subcutaneous ustekinumab.

## Case report

**Fig 1:**
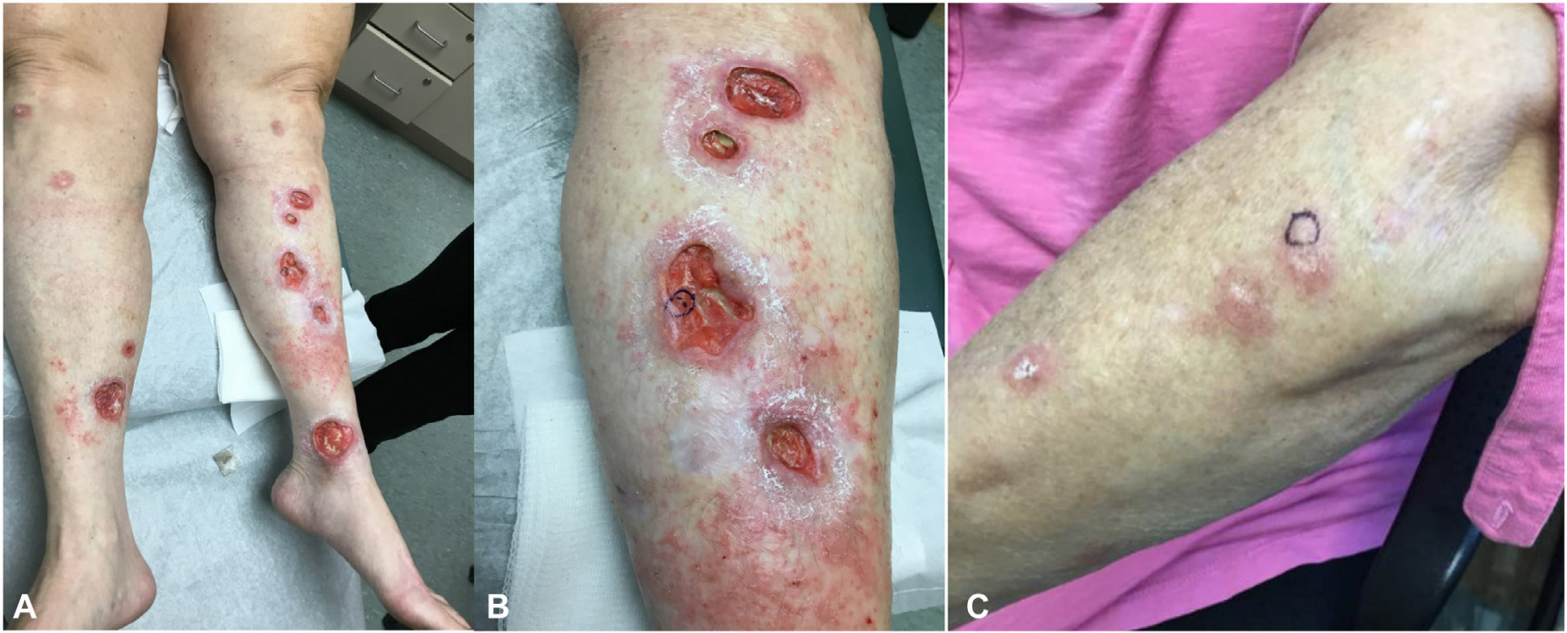
Clinical photographs of (**A, B**) lower extremities and (**C**) left forearm on presentation to the dermatology clinic. The location of punch biopsies for histopathologic evaluation on the (**B**) lower extremity and (**C**) forearm are depicted.

A 56-year-old White woman presented with a 6-year history of multiple persistent and recurrent erythematous papules/nodules, plaques, and ulcerations on her abdomen and bilateral upper and lower extremities (Fig 1). Her skin lesions started on her hand and legs but later progressed further onto her upper extremities and trunk. Her lesions were initially managed by rheumatology and wound care under the presumptive working diagnosis of antineutrophilic cytoplasmic antibody positive (ANCA+) vasculitis based on previous indirect immunofluorescence myeloperoxidase (pANCA+) serology (1:640) (serine protease 3 [cANCA] negative) as well as punch biopsies of the hand and leg demonstrating GD composed of lymphocytes, neutrophils, and eosinophils with bands of necrobiosis but no histologic evidence of vasculitis. She denied any medical or family history of diabetes mellitus (HbA1c 5.5 at presentation); glucose intolerance; or autoimmune disorders, including systemic lupus erythematosus, rheumatoid arthritis, Sjogren disease, or sarcoidosis.

**Fig 2:**
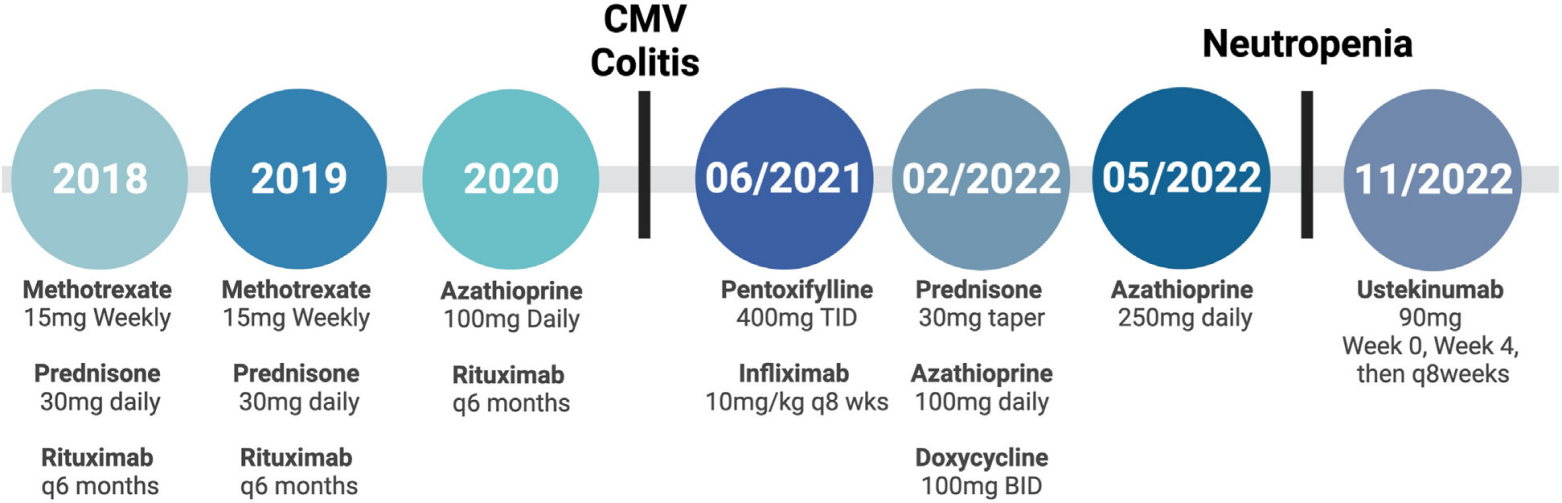
Representative timeline of patient’s treatment course for ulcerative lesions. *CMV*, Cytomegalovirus. Created with Biorender.com.

**Fig 3:**
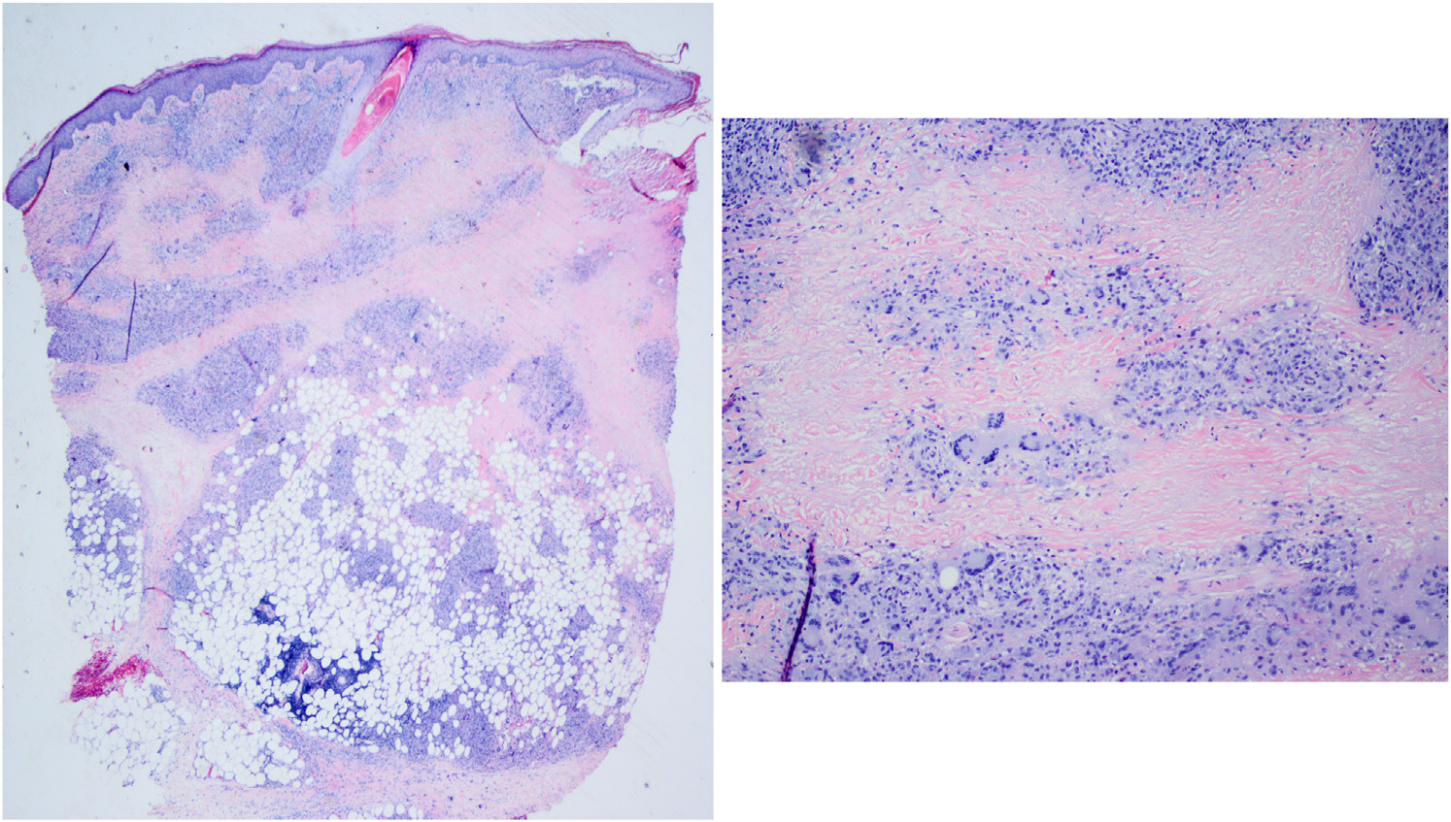
Representative images of low- and high-powered (200×) hematoxylin-eosin–stained punch biopsy obtained from the left forearm demonstrating granulomatous inflammation composed of histiocytes and multinucleated giant cells with associated lymphocytic inflammation and intervening bands of necrobiosis. (Left and right, Hematoxylin-eosin stain; original magnification: low-powered, ×20; high-powered, ×200).

She presented to dermatology 2 years ago for further recommendations after failing treatment with multiple agents (Fig 2), including medium- and high-potency topical steroids, oral prednisone (30 mg daily), methotrexate (15 mg weekly), rituximab (1 g every 6 months), and azathioprine (100 mg daily). She had some improvement with rituximab and azathioprine; however, her treatment course was complicated by severe cytomegalovirus colitis. Repeat biopsies of the lesions on the forearm and leg revealed a focally acanthotic epidermis with an overlying orthokeratotic scale. Bands of granulomatous inflammation composed of histiocytes, multinucleated giant cells, and lymphocytes were appreciated within the dermis in conjunction with intervening bands of necrobiosis (Fig 3). No vasculitis, frank caseation necrosis, or cholesterol clefts were appreciated on repeat biopsies. Alcian blue stain did not reveal an increase in mucin. Tissue culture was negative for bacterial, fungal, and mycobacterial infection. Additionally, repeat autoimmune serologic work-up was negative for myeloperoxidase (pANCA) and serine protease 3 (cANCA) antibodies by ELISA. Given these findings, a diagnosis of ulcerative NL was established.

**Fig 4:**
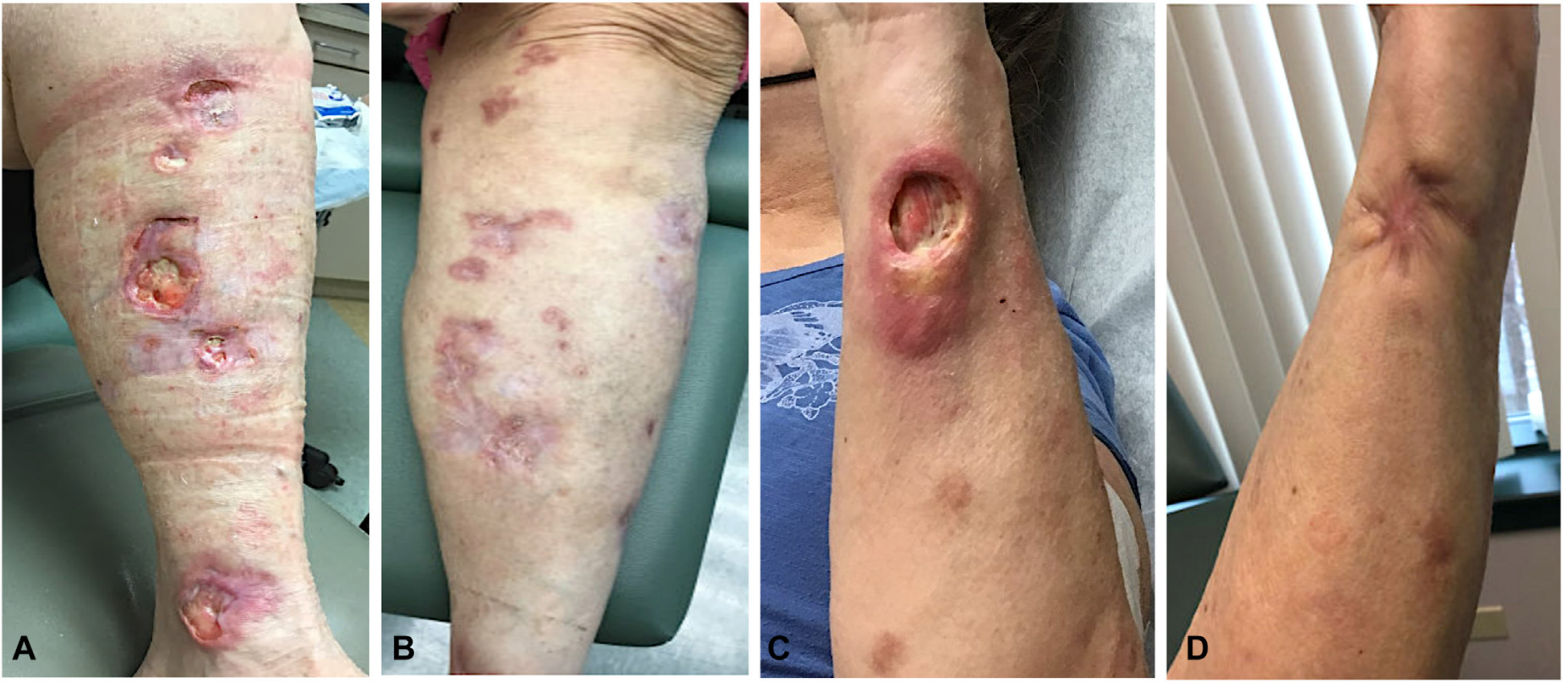
Serial clinical photographs of (**A, B**) left leg and (**C, D**) left forearm both (**A, C**) before treatment and (**B, D**) 4 months following ustekinumab treatment.

Treatments with oral pentoxifylline (400 mg 3 times daily) and infliximab infusions (10 mg/kg every 8 weeks) were unsuccessful. She was then restarted on azathioprine (100 mg daily), doxycycline (100 mg twice daily for 2 months), and an oral prednisone taper (30 mg with 10 mg taper each week for 3 weeks). After 2 months, azathioprine was increased to 250 mg daily and she exhibited mild improvement in wound closure and cosmesis of her lesions on the left forearm and lower extremities. However, she continued to develop new lesions on her bilateral upper extremities and maintained 10 to 15 active ulcerated lesions on average. Azathioprine was discontinued after 8 months because of new-onset neutropenia that improved after azathioprine discontinuation. She was then started on ustekinumab (90 mg subcutaneous [patient >100 kg] at week 0, week 4, and then every 8 weeks thereafter). At 4 months of follow-up, she had a near-complete resolution of the old active lesions with no new lesions and no reported side effects from ustekinumab (Fig 4). She only had one active healing lesion on her left arm.

## Discussion

The histopathologic term GD is broad, with significant variability in the microscopic findings. GD can be found in multiple cutaneous diseases, including NL, necrobiosis xanthogranuloma, rheumatoid arthritis, sarcoidosis, inflammatory bowel disease, and medium-vessel vasculitis.^1^^,^^2^ In this case, the patient was initially diagnosed with cutaneous manifestations secondary to ANCA+ medium-vessel vasculitis based on histologic findings of GD without vasculitis but with pANCA+ antibodies on serology. After multiple years of treatment, repeat skin biopsies from recalcitrant lesion sites showed histologic features of GD, but repeat serology autoantibody panel was negative for pANCA or cANCA that ultimately led to the refined diagnosis of NL. This case highlights the broad differential diagnosis associated with GD and that the definitive diagnosis for this case required a correlation between clinical history, histopathology of initial and larger repeat biopsies, and serologies. This case also highlights that ANCA serology by indirect immunofluorescence can have high false-positive rates because of myriad of potential concurrent rheumatologic or infectious etiologies, and recent guidelines advise consideration of ANCA serologies only following positive histologic evidence of vasculitis.^3^

The recalcitrant nature of these ulcerated lesions despite aggressive medical management with multiple immunosuppressants led us to the use of ustekinumab, a monoclonal antibody that functions as an antagonist to interleukin 12 (IL-12) and IL-23. Preclinical studies have shown both IL-12 and IL-23 contribute to chronic wound progression and ulcerations by serving as chemoattractants for tissue-resident proinflammatory macrophages and Th17 cells to active skin lesions and further lead to granuloma formation and collagen degeneration within the dermis.^1^^,^^4^, ^5^, ^6^ Furthermore, there has been a growing collection of cases series highlighting a therapeutic role for targeting IL-12/IL-23 using ustekinumab in cutaneous ulcerative and granulomatous dermatoses, including metastatic Crohn’s and pyoderma gangrenosum.^7^ We, therefore, hypothesized ustekinumab could be utilized as a therapeutic option to improve ulcer healing in the setting of NL by inhibiting this pathophysiologic mechanism. Over the last 4 months, there was sustained clinical improvement of this patient’s lesions, and she has not developed any new lesions. To our knowledge, this is the first reported case of severe disseminated ulcerative NL involving both upper and lower extremities that has been successfully treated using ustekinumab. Our observations align with previous cases demonstrating successful treatment of moderate-ulcerative NL limited to the lower extremities with ustekinumab (Table I).^8^, ^9^, ^10^ These findings underscore the involvement of IL-12 and IL-23 in the pathogenesis of NL and warrant further investigation into these cytokines’ roles in other forms of GD. In conclusion, this case highlights the potential utility of ustekinumab in the management of refractory cases of ulcerative NL.

**Table I:** Clinical characteristics of reported refractory necrobiosis lipoidica cases treated with ustekinumab

Patient demographic	Prior therapies	Ustekinumab dosing	Observed clinical response	Reference
Woman aged 42 y with no history of diabetes or glucose intolerance	Pentoxifylline Cyclosporine Mycophenolate mofetil Etanercept Adalimumab Infliximab and methotrexate Unna boot	45 mg subcutaneous Every 9 wks	1) Reduced pain and pruritus of NLD lesion 2) Absence of recurrent ulcerations for 6 mo posttreatment	Hassoun et al [8]
Woman aged 24 y with diabetes mellitus type I	Topical clobetasol propionate Intralesional triamcinolone doxycycline	45 mg subcutaneous Wk 0, 4, every 12 wks thereafter	Near-complete regranulation of NLD lesions within 12 wks	Beatty et al [9]
Woman aged 29 y with poorly controlled diabetes mellitus type II, tobacco use, hypertension and depression	Topical corticosteroids Topical tacrolimus Oral corticosteroids Intralesional corticosteroids Oral antibiotics Oral antifungals Hydroxychloroquine Pentoxifylline Adalimumab	90 mg subcutaneous Every 8 wks	Improvement of NLD lesion erythema and ulcerations	Pourang et al [10]

*NLD,* Necrobiosis lipoidica diabeticorum.

## Conflicts of interest

None disclosed.
